# Everolimus in combination with octreotide LAR in thymic atypical carcinoid

**DOI:** 10.1111/1759-7714.14887

**Published:** 2023-04-10

**Authors:** Tadashi Sakane, Tomoharu Nakano, Emi Hagui, Hiroshi Haneda, Katsuhiro Okuda

**Affiliations:** ^1^ Department of Thoracic Surgery Nagoya City University West Medical Center 1‐1‐1 Hirate‐cho, Kita‐ku Nagoya 462‐8508 Japan; ^2^ Department of Thoracic and Pediatric Surgery Nagoya City University Graduate School of Medical Sciences 1 Kawasumi, Mizuho‐cho, Mizuho‐ku Nagoya 467‐8601 Japan

**Keywords:** atypical carcinoid, everolimus, octreotide, thymic carcinoid

## Abstract

Thymic atypical carcinoids are extremely rare tumors and have a poor prognosis owing to their aggressive clinical course. The efficacy of treatments other than complete surgical resection is unclear. We herein report a postoperative recurrent case of thymic atypical carcinoid treated with everolimus and octreotide long‐acting repeatable (LAR). A 75‐year‐old woman was admitted to our department because a nodule was detected in the right lobe of thymus by annual computed tomography. The patient underwent thymothymectomy, and a diagnosis of thymic atypical carcinoid was made. One year and seven months after surgery, she developed multiple metastases in the lung, hilar and mediastinal lymph nodes, liver, and bone. Everolimus 10 mg/day was administered; however, the dose had to be reduced to 5 mg/day due to grade 3 hyperglycemia and grade 3 interstitial lung disease. Metastatic lesions other than liver metastasis markedly responded to everolimus, although the liver metastases gradually progressed. Three years and six months after surgery, she was administered octreotide LAR 30 mg per month in combination with everolimus. She has maintained stable disease for 8 months after the application of this combination therapy.

## Graphical abstract

Currently, only a few reports have described systemic antitumor therapy for thymic carcinoid. We herein report a postoperative recurrent case of thymic atypical carcinoid treated with everolimus and octreotide long‐acting repeatable (LAR).

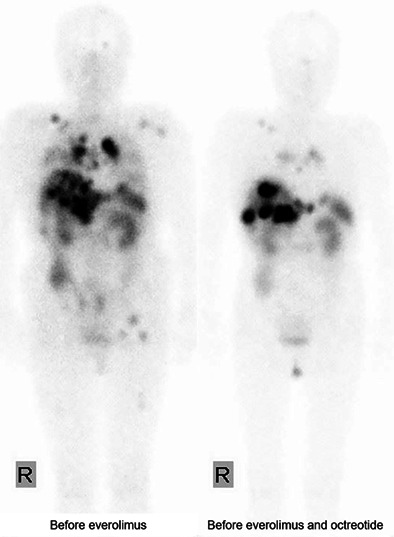

## INTRODUCTION

Thymic neuroendocrine tumors (NETs), first reported by Rosai and Higa in 1972, are rare malignant tumors accounting for less than 5% of all anterior mediastinal neoplasms.^1^, ^2^ Atypical carcinoids are an extremely rare type of NET with a poor prognosis owing to their invasive behavior, distant metastasis, and high rate of postoperative recurrence. Substantial evidence regarding the efficacy of chemotherapy is lacking.

We herein report a recurrent case of thymic atypical carcinoid treated with everolimus and octreotide long‐acting repeatable (LAR).

## CASE REPORT

A 75‐year‐old woman was admitted to our department due to a mediastinal mass detected by computed tomography (CT) at annual follow‐up. She had undergone mastectomy of the left breast plus axillary lymph node dissection for left breast cancer 13 years earlier. Three years after surgery, she developed multiple bone and subcutaneous metastases. She was started on anastrozole 1 mg per day, and the metastatic lesions shrank significantly.

**FIGURE 1:**
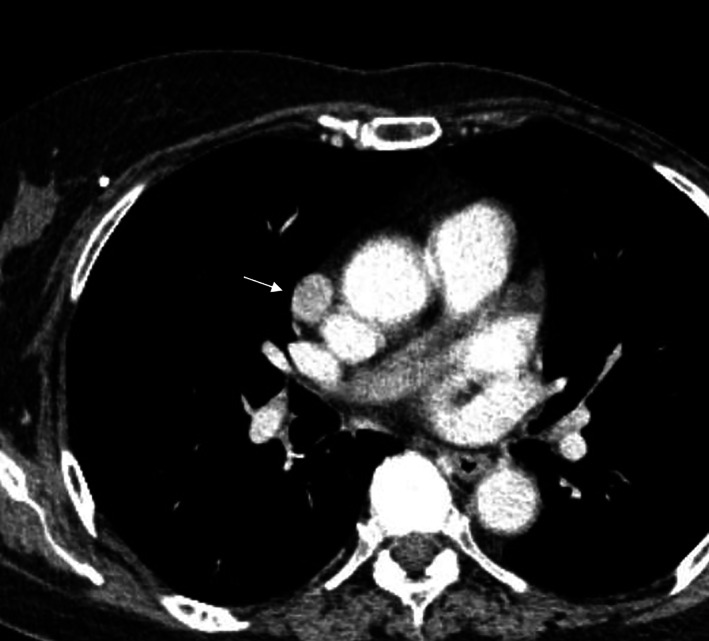
Contrast‐enhanced chest computed tomography revealed a well‐circumscribed tumor in the right lobe of the thymus (white arrow).

Chest CT on admission revealed a 2.0‐cm well circumscribed tumor in the right lobe of the thymus (Figure 1). No positive signs were detected on routine physical examination, and tumor markers were normal. Based on the clinical and radiological appearances, the patient was suspected of having thymoma and underwent thymothymectomy with thoracoscopic surgery via the subxiphoid approach.

**FIGURE 2:**
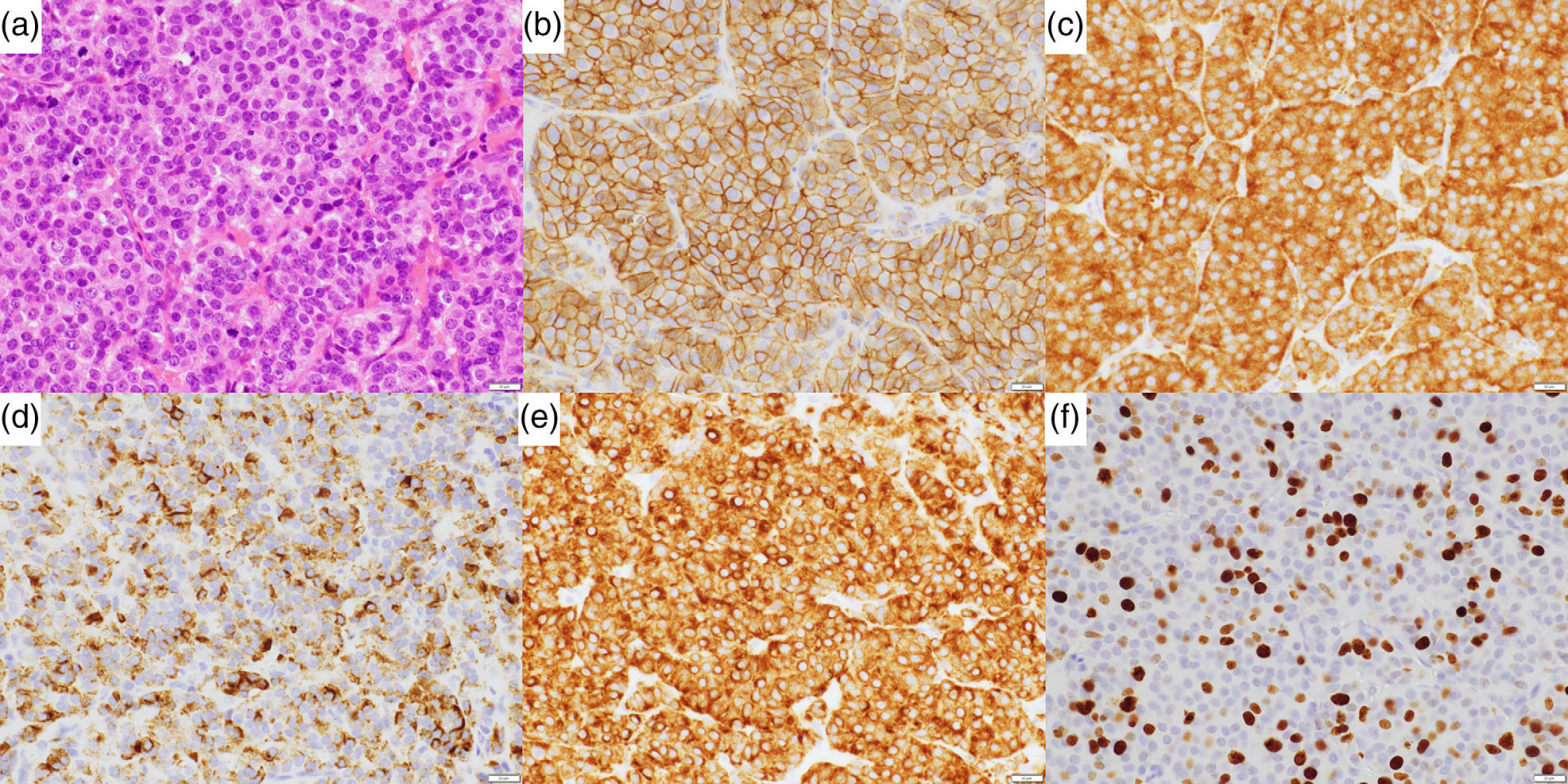
(a) A hematoxylin and eosin‐stained specimen showed small, round cells with nuclear pleomorphism and small nucleoli. (b) An immunohistochemical analysis demonstrated positivity for CD56, (c) synaptophysin, (d) chromogranin A, and (e) pan‐cytokeratin. (f) The staining index for Ki‐67 was 10%.

The postoperative course was uneventful. Macroscopically, the lesion was a well circumscribed tumor 1.7 × 1.4 cm in size with a soft, fleshy, and pale tan‐white cut surface. A histopathological examination showed that the tumor consisted of small‐size round cells with nuclear pleomorphism and small nucleoli, arranged in nests, lobules, and trabeculae with 10 mitoses per 10 high‐power fields and no necrosis (Figure 2). Immunohistochemically, the tumor cells were positive for cluster of differentiation 56 (CD56), synaptophysin, chromogranin A, and pan‐cytokeratin (AE1/3) but negative for thyroid transcription factor‐1, p63, CK5/6, c‐kit, and CD5, and the staining index for Ki‐67 was 10% (Figure 2). A diagnosis of thymic atypical carcinoid was thus established.

**FIGURE 3:**
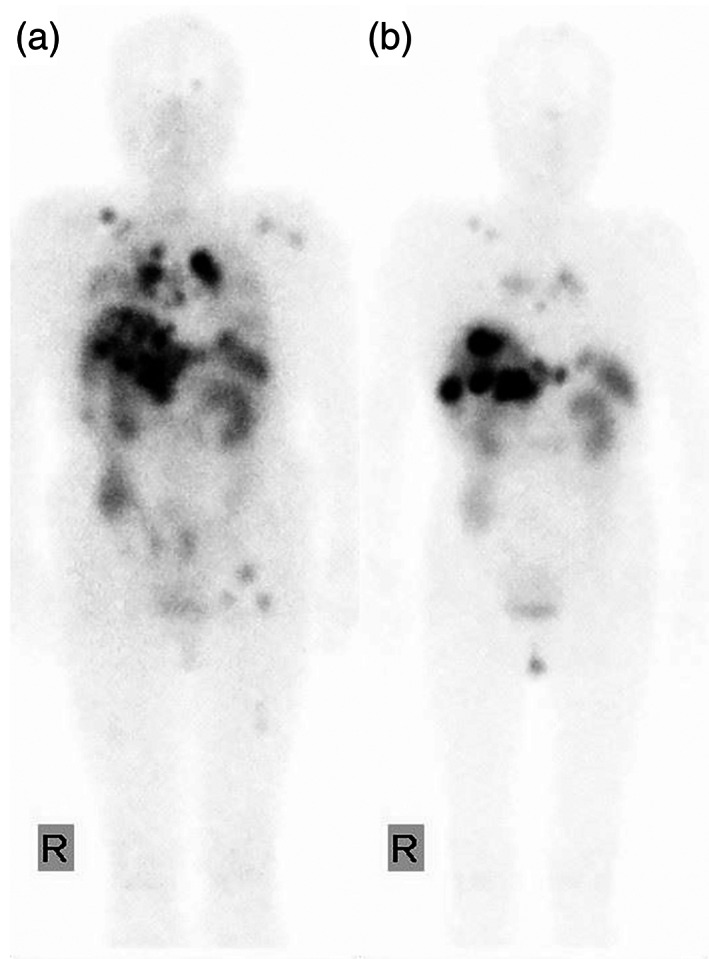
(a) Somatostatin receptor scintigraphy revealed an increased uptake in the lung, hilar and mediastinal lymph nodes, liver, and bone 1 year and 7 months after resection of thymic atypical carcinoid. (b) Metastatic lesions other than liver metastasis markedly responded to everolimus, leading them to be unidentifiable on somatostatin receptor scintigraphy.

As complete resection with microscopically negative margins was achieved, she received no adjuvant therapy. However, 1 year and 7 months after resection of thymic atypical carcinoid, multiple metastases in the lung, liver, and bone were found on CT. Positron emission tomography revealed a high uptake in those lesions. Somatostatin receptor scintigraphy (SRS) revealed an increased uptake in the above‐mentioned lesions and hilar and mediastinal lymph nodes (Figure 3). Subsequently, everolimus 10 mg/day was administered with a diagnosis of multiple metastasis of atypical carcinoid.

However, the patient developed grade 3 hyperglycemia and grade 3 interstitial lung disease 1 month after the application of everolimus. The dose of everolimus had to be reduced to 5 mg/day due to these drug‐induced adverse events. Metastatic lesions other than liver metastasis markedly responded to everolimus, leading them to be unidentifiable on SRS, although the liver metastases gradually progressed (Figure 3). Three years and 6 months after surgery, the patient was administered octreotide LAR at 30 mg/month in combination with everolimus. Stable disease was then maintained in this patient for 8 months after the application of combination therapy.

## DISCUSSION

There are no trial‐proven therapeutic strategies in thymic NETs due to the rarity of this disease. Therapeutic protocols are thus adopted from NETs of other origin. According to the current ESMO guidelines on lung and thymic carcinoids, based on the very limited data available, surgery is recommended for thymic carcinoids deemed radically resectable.^3^ In addition, this guideline suggests no benefit from routine adjuvant therapy after R0 resection, although postoperative therapies should be discussed individually in patients with advanced‐stage R0 or R1–2 resection.^3^ The therapeutic options for inoperable or metastatic disease include everolimus, somatostatin analogs (SSAs), chemotherapy, peptide receptor radionuclide therapy, and interferon‐α, although the efficacy of these treatments is not satisfactory. Among these approaches, everolimus and SSAs are recommended as the first line in case of thymic atypical carcinoid.

Everolimus is an oral inhibitor of the mammalian target of rapamycin pathway, approved as the first agent for therapy of pulmonary NETs based on the results of the phase III RADIANT‐4 study.^4^ In recent years, strong evidence has emerged of an antiproliferative effects of SSAs, including octreotide and lanreotide, on NETs, which is thought to occur via direct or indirect mechanisms.^5^ Everolimus and SSAs both display favorable outcomes in patients with NETs, but the efficacy and safety of their use in combination are still uncertain. Several studies, including systematic reviews, support the use of this combination therapy, and this strategy is widely used for gastroenteropancreatic NETs in clinical practice, although current guidelines do not recommend combination therapy for lung and thymic NETs.^6^, ^7^, ^8^, ^9^

In thymic carcinoids, only a few cases have been reported that were treated with everolimus, octreotide, pasireotide, sunitinib, or chemotherapy.^10^, ^11^, ^12^, ^13^, ^14^ To our knowledge, this is the first thymic atypical carcinoid case treated with the combination of everolimus and SSAs. Everolimus and SSAs commonly induce different adverse events. In the present case, the patient suffered everolimus‐induced grade 3 hyperglycemia and grade 3 interstitial lung disease, although a reduced dose of 5 mg daily for everolimus in combination with octreotide has not induced significant adverse events.

In summary, we describe for the first time a particularly rare case of thymic atypical carcinoid, in which postoperative recurrence has been managed with everolimus plus octreotide LAR. We believe that the accumulation of more cases will lead to the development of effective therapy for this disease.

## AUTHOR CONTRIBUTIONS

TS, HH, and KO made substantial contributions to the conception of the study. TS and KO drafted the original manuscript. TN, EH, HH, and KO reviewed the manuscript draft and revised it critically for intellectual content. All authors approved the final version of the manuscript to be published.

## CONFLICT OF INTEREST STATEMENT

The authors have no conflicts of interest to declare.
